# DSHformer: Locality-Sensitive Hash Attention and Prototype Alignment for Sensor-Based Human Activity Recognition

**DOI:** 10.3390/s26123803

**Published:** 2026-06-15

**Authors:** Xiaofeng Zhang, Muzi Ding, Tangzhi Teng, Jie Wan, Hong Ding

**Affiliations:** School of Artificial Intelligence and Computer Science, Nantong University, Seyuan Campus, Nantong 226019, China; ntuzxf@163.com (X.Z.); dingmuzi@ntu.edu.cn (M.D.); tengtangzhi@ntu.edu.cn (T.T.); jiewan@ntu.edu.cn (J.W.)

**Keywords:** human activity recognition, wearable sensors, Transformer, locality-sensitive hashing, domain shift, prototype alignment

## Abstract

**Highlights:**

**What are the main findings?**

- We introduce DSHformer, which combines patch-based channel–temporal feature extraction, prototype-guided feature refinement, and decomposition-guided LSH-based attention for sensor-based HAR.
- Extensive experiments on five public benchmarks show that DSHformer achieves competitive or superior performance compared with several HAR baselines and recent Transformer variants with attention-level efficiency advantages that become pronounced on longer sequences.

**What are the implications of the main findings?**

- This paper demonstrates that strong HAR performance does not necessarily require heavy full-attention architectures.
- This paper offers a practical framework for balancing recognition accuracy, model compactness, and attention-level efficiency on longer wearable-sensor sequences.

**Abstract:**

Sensor-based human activity recognition (HAR) plays a fundamental role in healthcare monitoring, sports analytics, and ambient-assisted living. Although deep learning has substantially advanced HAR performance, two practical issues still limit its real-world deployment: (i) the distribution shift caused by changes in users or sensor placements can degrade generalization, and (ii) the quadratic $O(L^{2})$ complexity of standard self-attention hinders efficient long-sequence modeling on resource-constrained wearable devices. To address these issues, we propose DSHformer, which is an accuracy-oriented HAR framework that combines compact channel–temporal encoding with locality-sensitive hashing (LSH)-based attention. Specifically, DSHformer (i) employs a low-parameter patch-based graph-attention encoder to jointly model latent relationships among sensor channel–temporal dynamics; (ii) introduces a trainable prototype pool together with a multi-layer decomposition network to improve intra-class compactness and inter-class separability via prototype alignment; and (iii) introduces a decomposition-stable LSH-based attention mechanism tailored for HAR, whose core design couples prototype-guided feature decomposition with locality-sensitive hashing to ensure that semantically related tokens remain consistently grouped in the same hash bucket even after decomposition-induced attenuation. The mechanism thereby operates at O(LlogL) attention complexity on longer sensor sequences. Extensive experiments on five public benchmarks (WISDM, UCI-HAR, PAMAP2, Opportunity, and UniMiB-SHAR) show that DSHformer achieves accuracies of 98.6%, 93.7%, 98.4%, 88.5%, and 96.6%, respectively, achieving competitive or superior performance compared with both Transformer variants and HAR-specific baselines under the adopted benchmark protocols. Ablation studies further confirm the complementary contribution of each component.

## 1. Introduction

Sensor-based human activity recognition (HAR) methods can identify and classify activities by analyzing sensor signals. Current trends indicate that wearable devices have become widely integrated into our lives, providing a foundation for smart home and transportation applications [1,2], ranging from rehabilitation and caregiving for the growing elderly population to improving human performance [3,4]. By embedding tiny sensors in wearable devices, we can automatically and accurately recognize human activities, which are fundamental to these applications.

Wearable technology creates multi-channel time-series data streams by continually recording measurement results through several sensor channels. Finding and categorizing the timing of actions within these produced time series is hence the HAR challenge. The system can identify activities and automatically extract characteristics from the raw sensor data by using deep learning networks, which increases the recognition accuracy [5,6,7].

### 1.1. Problem and Motivations

Although end-to-end deep learning architectures have made significant progress on HAR tasks, we identify three untapped dimensions that substantially limit the performance of current state-of-the-art frameworks:

HAR systems frequently encounter distribution shifts caused by changes in users, sensor placements, or device types when moving from training to real-world testing. Although transfer learning has been shown to improve generalization in computer vision tasks [8,9,10], these methods typically require access to target-domain data during training. In real-world wearable applications, however, retraining the model whenever users or devices change is impractical.

HAR data collection yields motion measurements across multiple sensors and channels, suggesting that the channel and temporal structure can jointly improve activity encoding. However, existing models often over-parameterize this process by stacking heavy convolutional or attention blocks to capture every possible inter-channel–temporal interaction. Reducing the number of trainable parameters while preserving representational capacity remains an open challenge [10,11].

Attention mechanisms allow models to focus on salient parts of the input, but the standard self-attention has $O(L^{2})$ complexity, which is impractical for long sensor windows on resource-constrained wearable devices. Although several efficient Transformer variants reduce this cost, they often sacrifice recognition accuracy in doing so [12,13].

### 1.2. Our Contributions

The main contributions of this paper are summarized as follows:

- **Low-parameter channel–temporal feature encoder.** We design a compact yet expressive feature extractor that jointly models inter-channel correlations and temporal dependencies. Specifically, we first apply stacked 1D convolutions to capture local temporal patterns, then partition the sequence into non-overlapping patches and build a patch-wise channel graph to encode cross-sensor relations, and finally refine the patch representations with an attention-augmented LSTM. The entire encoder uses only seven convolutional layers, two fully connected layers, and a single LSTM layer, achieving strong representation capability with markedly fewer trainable parameters than existing Transformer-based HAR models (see Section 3.2).
- **Prototype-based alignment for robust representations.** To improve intra-class compactness and inter-class separability under varying data conditions, we construct a trainable prototype pool whose entries represent class-wise reference samples and stack a multi-layer decomposition network that separates similar and dissimilar components between inputs and prototypes. An inter-class margin loss further enlarges the separation between prototypes of different activities, yielding more compact and class-discriminative representations (see Section 3.3 and Section 3.4).
- **Decomposition-guided LSH-based attention for HAR.** We replace the standard self-attention with a decomposition-guided LSH-based attention mechanism tailored for multi-channel sensor sequences. By coupling prototype-guided feature decomposition with locality-sensitive hashing, DSHformer ensures that semantically related temporal tokens remain grouped in the same hash bucket even after decomposition-induced feature attenuation, and it computes attention only among those grouped tokens. The mechanism operates at O(LlogL) attention complexity—the same asymptotic class as Reformer—while the novelty lies in the HAR-tailored decomposition-stable hashing design rather than the complexity class itself.
- **Comprehensive empirical validation.** We evaluate DSHformer under a unified protocol on five widely used HAR benchmarks—WISDM, UCI-HAR, PAMAP2, Opportunity, and UniMiB-SHAR—where it achieves competitive or superior performance compared with both HAR-specific baselines and recent Transformer variants. Extensive ablation and visualization studies further verify the individual contribution of each proposed component (see Section 4.3 and Section 4.4).

## 2. Related Work

### 2.1. Deep Learning for Human Activity Recognition

By making it possible to automatically extract intricate features from unprocessed sensor data, deep learning has completely transformed HAR [14]. The intricacy of human motion cannot be fully captured by traditional approaches, which frequently rely on manual feature extraction [15]. On the other hand, discriminative representations can be learned directly from data using deep learning models like Convolutional Neural Networks (CNNs) and Recurrent Neural Networks (RNNs) [16]. Accurate activity recognition depends on the spatial and temporal connections that are encoded by these representations. These models’ performance in practical applications has also been improved by the abundance of data sources made available by the growth of wearable technology and smart home technologies. This has led to a major development in the industry with deep learning-based HAR systems currently being widely employed in domains including intelligent environment control and health monitoring.

Emerging deep learning techniques have recently shown remarkable results in a variety of research fields, including speech recognition, natural language processing, and traffic flow prediction [17]. Deep learning has made it possible for automatic end-to-end feature extraction in sensor-based human activity identification (HAR), greatly minimizing the need for laborious feature engineering. These developments have led to the widespread and effective use of deep learning techniques in a variety of HAR application settings [18,19,20].

The capacity of the model to capture both short-term and long-term dependencies has been improved by the recent widespread usage of deep learning architectures based on time-series decomposition to deconstruct and reconstruct temporal patterns from complicated time-series data [11,21,22,23]. In order to capture important activity aspects, these studies frequently use multi-scale convolution to break down time-series data into trend and seasonal components. Classification networks are then used to integrate and link the retrieved latent data to activity categories. Wu et al. [12,13,24], for instance, presented a Fourier transform-based time-series decomposition architecture that uses main frequency results to break down time-series data into several periodic patterns.

Furthermore, in deep learning, channel and temporal encoding have emerged as crucial methods for managing intricate time-series data [16,25]. Through their ability to capture spatial information and temporal dependencies, they greatly improve the performance of models in a variety of dynamic tasks. We previously proposed a deep learning method based on spatio-temporal encoding [26], which uses a two-stage graph network to model the spatial patterns of multivariate time-series data and creates an attention-based neural control differential equation network to capture dynamic dependencies across time steps. Even while our previous work, as outlined in Section 1.1, produced some results, the current research tackles a more specialized and intricate issue that calls for novel approaches. In contrast to earlier research, this paper suggests a more effective and parameter-efficient encoding structure while delving deeper into the use of channel and temporal encoding in HAR.

### 2.2. Transformer-Based Methods

Since the Transformer was introduced by Vaswani et al. [7], self-attention has been widely adopted in time-series modeling to capture long-range dependencies.

However, a significant obstacle to directly applying Transformers from NLP to time-series jobs is the Transformer architecture’s quadratic time and memory complexity. This intricacy limits the model’s usefulness by causing a dramatic increase in memory and processing resources when working with lengthy sequences. Researchers have put out a number of computationally effective time-series Transformer models in an effort to address this problem. By altering the self-attention mechanism, these models achieve more efficient processing while lowering computational complexity. For instance, by using a variety of techniques like sparse attention matrices, divide-and-conquer tactics, or adding more inductive biases, models like Informer [27], ETC [28], Pyraformer [29], and FEDformer [30] have successfully reduced complexity to linear or near-linear levels (such as O(L) or O(LlogL)). In addition to improving the model’s capacity to manage lengthy sequences, these enhancements offer fresh avenues for investigation and techniques in the time series analysis community.

Locality-Sensitive Hashing (LSH) provides an efficient approximate nearest-neighbor mechanism by mapping similar high-dimensional vectors into the same or nearby hash buckets. This property has motivated LSH-based attention mechanisms such as Reformer [5] where attention is restricted to tokens with similar hash assignments.

While Transformer-based deep learning frameworks have demonstrated significant promise in managing multi-channel time-series data, there are still issues with striking a balance between complexity and accuracy. Optimizing model complexity while preserving high performance is a crucial problem that requires more research in this area, because high accuracy frequently entails greater model complexity.

## 3. Materials and Methods

All experiments used publicly available datasets and did not involve additional physical data-collection equipment. The computational experiments were implemented in Python 3.13.5 using NumPy 2.3.0 and PyTorch 2.11.0+cu128 with CUDA 12.8; scikit-learn 1.7.2 was used for evaluation metrics and t-SNE visualization, and pandas 2.3.0 and SciPy 1.17.1 were used for data processing. The experiments were run on a computer equipped with an Intel UHD Graphics adapter and an NVIDIA GeForce RTX 4060 Laptop GPU.

**Problem definition.** First, we provide a formal definition of the human activity recognition (HAR) task. Let $D={\left\{\left(X^{\left(i\right)},y^{\left(i\right)}\right)\right\}}_{i=1}^{N}$ denote a labeled HAR dataset containing *N* samples. Each sample $X^{\left(i\right)}\in R^{M\times T}$ is a multivariate sensor time series where *M* denotes the number of sensor channels or variables and *T* denotes the number of temporal steps within one sample window. The vector at time step *t* is denoted as $x_{t}^{\left(i\right)}={\left[x_{1,t}^{\left(i\right)},x_{2,t}^{\left(i\right)},\ldots ,x_{M,t}^{\left(i\right)}\right]}^{⊤}\in R^{M}$, where t=1,…,T. The corresponding activity label is $y^{\left(i\right)}\in \Omega =\left\{1,2,\ldots ,K\right\}$ where *K* is the number of activity classes. The goal of HAR is to learn a classifier $f_{\theta }:R^{M\times T}\to \Omega$ that predicts the activity label of an unseen multivariate sensor sequence.

### 3.1. Model Architecture Overview

**Figure 1:**
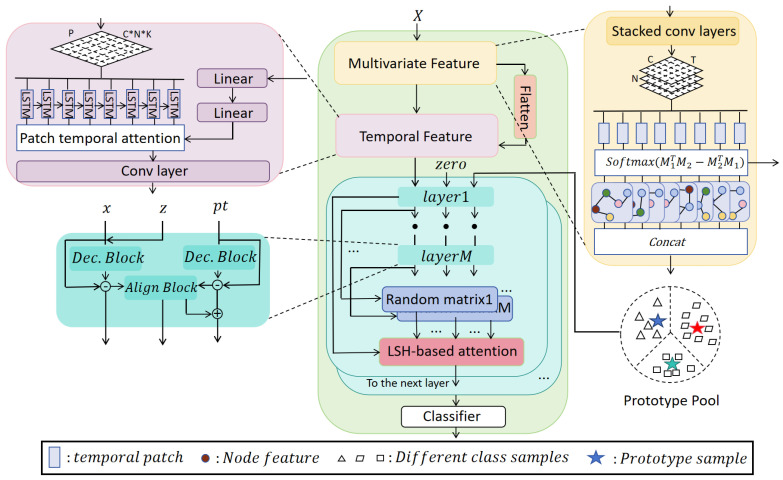
Overall architecture of DSHformer, including patch-based channel–temporal feature extraction, prototype-guided decomposition and alignment, and LSH-based attention. Arrows indicate data flow, colored dots denote graph node features, stars denote prototype samples, and triangles, parallelograms, and squares denote samples from different classes.

As shown in Figure 1, the input *X* is first processed by a channel–temporal feature encoder. Stacked 1D convolutional layers capture abstract local temporal patterns from neighboring time steps without yet fusing information across channels. The sequence is then partitioned along the temporal dimension into non-overlapping patches, and a channel-wise graph matrix is constructed for each patch to model inter-sensor dependencies. The channel-fused patch representations are fed into a single-layer LSTM that treats each patch as a temporal token, and a patch-level attention matrix, computed from the similarity between graph matrices, reweights the LSTM outputs to emphasize informative patches.

In parallel, a trainable prototype pool P stores class-specific reference samples. Both X and P are fed into a multi-layer decomposition network, where Decomposition Blocks (Dec Blocks) separate similar and dissimilar components between inputs and prototypes, and alignment blocks reduce the distribution gap between training and test samples. Random projection matrices then map the decomposed features into hash buckets, and attention weights are computed only among time steps that share the same hash code—so that features with similar temporal semantics remain grouped even after decomposition-induced attenuation.

**Figure 2:**
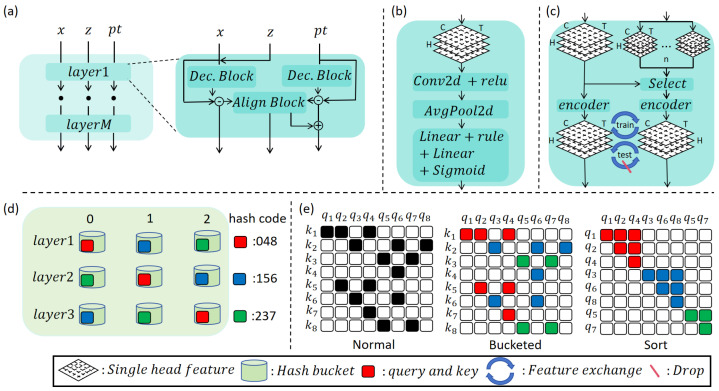
Decomposition -guided LSH-based attention module. (**a**) Multi-layer decomposition and alignment framework; (**b**) hash-code generation block; (**c**) prototype-guided feature exchange and selection; (**d**) hash-bucket assignment across layers; (**e**) normal attention, bucketed attention, and sorted bucketed attention. Black framed modules denote repeated decomposition/alignment operations, cylinders denote hash buckets, colored squares denote hash codes, circular arrows denote feature exchange, and the slash marker denotes dropped features.

Overall, the model (i) extracts channel and temporal features with few trainable parameters and linear-time encoding complexity; (ii) applies a decomposition-guided LSH-based attention mechanism that maintains semantic token groupings and operates at O(LlogL) attention complexity; and (iii) maintains a trainable prototype pool that encourages class-discriminative and compact representations. This two-stage refinement yields rich feature representations prior to classification. Each component of Figure 1 and Figure 2 is described in detail in the following sections.

### 3.2. Patch-Based Graph Attention and Temporal Feature Learning Module

The fundamental principle behind the approach that combines the temporal decomposition network with the Locality-Sensitive Hashing (LSH) algorithm presented in Section 3.3 is that similar temporal features can be assigned to the same hash bucket even when they are in different decomposition states. As a result, we must create a feature extraction network that satisfies the following requirements. (i) The network must be able to aggregate similar characteristics across many variables and time steps, as the decomposition network necessitates continual attenuation of these features; (ii) the feature extractor should remain lightweight so that it does not dominate the computational cost before the LSH-based attention stage.

As the main feature extractor, we first use a multi-layer stacked convolutional network [16]. Four convolutional layers are used to process the input sensor sequence, and each layer combines ReLU nonlinear activation with 64 one-dimensional convolutional filters of size 5 along the temporal dimension. These stacked temporal convolutions extract local motion patterns from the raw sensor sequence while preserving the channel-wise structure required for subsequent graph-based modeling.

We then construct a channel feature extraction network because graph structures have been widely used to model relationships among different positions or variables in structured data [31,32,33]. After the temporal convolutional transformation, the feature map is partitioned into non-overlapping patches along the temporal dimension. This patching operation provides local temporal segments for constructing channel-wise graph representations:

(1) $$\begin{array}{cc} H & =\mathrm{Conv}1D(X),\\ r & =\left(n-(T\;\mathrm{mod}\;n)\right)\;\mathrm{mod}\;n,\\ H^{\mathrm{pad}} & ={\mathrm{Concat}}_{t}\left(H,0_{B\times C_{h}\times r\times M}\right),\\ N_{p} & =\frac{T+r}{n}. \end{array}$$

where X denotes the input sensor sequence, H denotes the convolutional feature map, and $H^{\mathrm{pad}}$ denotes the temporally padded feature map. *B* is the batch size, $C_{h}$ is the number of convolutional hidden channels, *M* is the sensor-channel dimension, *T* is the original sequence length, *n* is the patch length, *r* is the temporal padding length, and $N_{p}$ is the number of temporal patches. The padding vector $0_{B\times C_{h}\times r\times M}$ is concatenated along the temporal dimension. When *T* is divisible by *n*, r=0, and no temporal padding is added.

For the *i*-th sample and the *p*-th temporal patch, let $u_{i,p}^{m}$ denote the feature vector associated with the *m*-th sensor channel. We compute the channel-to-channel affinity within this patch as

(2) $$\begin{array}{cc} \alpha_{i,p}^{m,m^{\prime }} & =\frac{\exp\left(\phi {\left(u_{i,p}^{m}\right)}^{⊤}\psi \left(u_{i,p}^{m^{\prime }}\right)\right)}{\sum_{\ell =1}^{M}\exp\left(\phi {\left(u_{i,p}^{m}\right)}^{⊤}\psi \left(u_{i,p}^{\ell }\right)\right)}, \end{array}$$

(3) $$\begin{array}{cc} {\hat{u}}_{i,p}^{m} & =u_{i,p}^{m}+\gamma \sum_{m^{\prime }=1}^{M}\alpha_{i,p}^{m,m^{\prime }}u_{i,p}^{m^{\prime }}. \end{array}$$

For each temporal patch, the channel affinity scores form a channel relation matrix

(4) $$A_{i,p}={\left[\alpha_{i,p}^{m,m^{\prime }}\right]}_{m,m^{\prime }=1}^{M}\in R^{M\times M}.$$

Stacking the channel-refined patch features over all $N_{p}$ patches gives

(5) $$E={\mathrm{Stack}}_{p=1}^{N_{p}}\left({\hat{U}}_{p}\right)\in R^{B\times N_{p}\times D_{e}},\;A={\mathrm{Stack}}_{i=1,p=1}^{B,N_{p}}\left(A_{i,p}\right)\in R^{B\times N_{p}\times M\times M}.$$

where $\alpha_{i,p}^{m,m^{\prime }}$ denotes the channel affinity weight from the *m*-th sensor channel to the $m^{\prime }$-th sensor channel in the *p*-th patch of the *i*-th sample. ϕ(·) and ψ(·) are learnable one-dimensional convolutional projections used to generate query and key features, respectively. ${\hat{u}}_{i,p}^{m}$ is the channel-refined representation obtained through residual graph-based aggregation, and γ is a learnable scaling parameter. $A_{i,p}$ is the M×M channel relation matrix for one temporal patch. After stacking all samples and patches, $A\in R^{B\times N_{p}\times M\times M}$, where *B* is the batch size and $N_{p}$ is the number of temporal patches. $E\in R^{B\times N_{p}\times D_{e}}$ denotes the patch-level feature tensor after local graph refinement and flattening, where $D_{e}$ is the patch-level feature dimension.

We further design a patch-level temporal feature extraction network because Recurrent Neural Networks are effective in modeling temporal dependencies [1,6,16]. The patch-level feature tensor $E\in R^{B\times N_{p}\times D_{e}}$ is fed into a single-layer LSTM to obtain a sequence of hidden states $H^{\mathrm{lstm}}=\left[h_{1}^{\mathrm{lstm}},h_{2}^{\mathrm{lstm}},\ldots ,h_{N_{p}}^{\mathrm{lstm}}\right]$. To incorporate the graph structure of each temporal patch, the channel relation matrix $A_{p}$ is flattened and mapped to a patch-level temporal weight:

(6) $$\begin{array}{cc} w_{p} & =\frac{\exp\left(\mathrm{fc}(\mathrm{flatten}\left(A_{p}\right))\right)}{\sum_{p^{\prime }=1}^{N_{p}}\exp\left(\mathrm{fc}(\mathrm{flatten}\left(A_{p^{\prime }}\right))\right)}, \end{array}$$

(7) $$\begin{array}{cc} h_{p}^{\mathrm{out}} & =\left(1+\gamma_{t}w_{p}\right)h_{p}^{\mathrm{lstm}},\;p=1,\ldots ,N_{p}. \end{array}$$

where $w_{p}$ denotes the temporal importance weight of the *p*-th patch, fc(·) denotes a linear mapping, flatten(·) denotes the flattening operation, $\gamma_{t}$ is a learnable scaling parameter, $h_{p}^{\mathrm{lstm}}$ is the LSTM hidden state of the *p*-th patch, and $h_{p}^{\mathrm{out}}$ is the temporally reweighted patch representation. The output sequence $H^{\mathrm{out}}=\left[h_{1}^{\mathrm{out}},h_{2}^{\mathrm{out}},\ldots ,h_{N_{p}}^{\mathrm{out}}\right]$ is used as the encoded temporal representation.

### 3.3. Decomposition Network and LSH-Based Attention

Using temporal information from different postures is important for fine-grained human activity recognition with wearable sensors. To improve sensor feature representation, we design a decomposition-guided LSH-based attention module that learns interactions between input temporal features and prototype features while avoiding the quadratic computational cost of full self-attention [5,7,29]. To avoid ambiguity, Locality-Sensitive Hashing (LSH) refers to the hashing strategy that maps similar temporal tokens into the same or nearby hash buckets, whereas LSH-based attention refers to the attention mechanism that computes local attention within the selected hash buckets. In this paper, the proposed decomposition-guided LSH-based attention module consists of three main components: a multi-layer temporal decomposition network, hash-code generation based on LSH, and local attention within hash buckets, corresponding to Figure 2a, Figure 2d and Figure 2e, respectively. First, we decompose the prototype features and the input temporal features into similar and dissimilar components:

(8) $$\begin{array}{cc} h_{s} & =\mathrm{sigmoid}\;\left(\mathrm{fc}\;\left(\mathrm{AvgPool}\;\left(\mathrm{Conv}(h+\beta \cdot z)\right)\right)\right)⊙h, \end{array}$$

(9) $$\begin{array}{cc} h_{ns} & =h-h_{s}, \end{array}$$

(10) $$\begin{array}{cc} p_{s}^{k} & =\mathrm{sigmoid}\;\left(\mathrm{fc}\;\left(\mathrm{AvgPool}\;\left(\mathrm{Conv}(p^{k})\right)\right)\right)⊙p^{k}, \end{array}$$

(11) $$\begin{array}{cc} p_{ns}^{k} & =p^{k}-p_{s}^{k}. \end{array}$$

where z denotes the initialized zero vector in the first layer of the temporal decomposition network; in subsequent layers, it represents the interaction features between the input samples and the aligned prototype samples. h denotes the hidden state of the input feature; $h_{s}$ and $h_{ns}$ denote its similar and dissimilar components, respectively. $p^{k}$ denotes the *k*-th prototype sample; $p_{s}^{k}$ and $p_{ns}^{k}$ denote its similar and dissimilar components, respectively. β is the attenuation coefficient.

The closest prototype sample is then determined by selecting the *i*-th prototype sample based on the ground-truth label during training or by finding the nearest prototype via Euclidean distance during inference. Then, we align and interact the input features with the selected prototype features. The interaction operation encourages the feature distribution of input samples to become closer to the corresponding prototype distribution, while the alignment operation provides a prototype reference for reducing the mismatch between training and inference representations.

(12) $$\begin{array}{cc} x_{\mathrm{ali}} & =\left(W\left(\mathrm{enc}\left(h_{s}\right)-\mathrm{enc}\left(p_{s}^{*}\right)\right)+b\right)⊙\mathrm{enc}\left(p_{s}^{*}\right)+h_{ns}, \end{array}$$

(13) $$\begin{array}{cc} p_{\mathrm{ali}} & =\left(W\left(\mathrm{enc}\left(p_{s}^{*}\right)-\mathrm{enc}\left(h_{s}\right)\right)+b\right)⊙\mathrm{enc}\left(h_{s}\right)+p_{ns}^{*}. \end{array}$$

where $x_{\mathrm{ali}}$ and $p_{\mathrm{ali}}$ denote the aligned input feature and the aligned prototype feature, respectively. enc(·) is a feature encoder comprising batch normalization, ReLU activation, and a Conv2D layer. W and b denote the weight matrix and bias vector of the linear transformation, respectively. $h_{s}$ and $h_{ns}$ denote the similar and dissimilar components of the input feature, respectively (see Equations (8) and (9)). $p_{s}^{*}$ and $p_{ns}^{*}$ denote the similar and dissimilar components of the selected prototype $p^{*}$, whose selection criterion is defined in Equation (14) below. ⊙ denotes element-wise multiplication.

The prototype pool is organized in a class-wise manner. Let $P=\{P_{1},P_{2},\ldots ,P_{K}\}$ denote the full prototype pool, where $P_{k}={\left\{p_{k,j}\right\}}_{j=1}^{J_{k}}$ is the prototype set of the *k*-th activity class. The prototype used for alignment is selected as

(14) $$p^{*}=\left\{\begin{array}{cc} p\in P_{y}, & \mathrm{during}\;\mathrm{training},\\ \arg\min_{p\in P}d\left(s,p\right), & \mathrm{during}\;\mathrm{inference}, \end{array}\right.$$

where *y* is the ground-truth label available during training, s denotes the decomposed feature used for prototype matching, and d(·,·) denotes the Euclidean distance.

During training, the class-wise prototype set associated with the ground-truth label is used. During inference, the ground-truth label is unavailable, and the nearest prototype is selected from the full prototype pool according to the Euclidean distance. Since bucket query and key counts can vary, we enforce $H\left(k_{j}\right)=H\left(q_{j}\right)$ by setting $k_{j}=\frac{q_{j}}{∥q_{j}∥}$ [5], where *j* indexes the token position, $q_{j}\in R^{d}$ is the query vector at position *j*, and $k_{j}$ is its $\ell_{2}$-normalized key. The queries are then sorted in two levels: first by bucket number and then by sequence position within each bucket. As seen in Figure 2e, sorting yields a rearranged attention matrix with queries in the same bucket clustered near the diagonal. First, a unique combination identifier is created for each query at position (a,t) by combining its bucket assignment and sequence position:

(15) $${\mathrm{bp}}_{a,t}=N_{p}\times b_{a,t}+\left(\tau_{a,t}\;\mathrm{mod}\;N_{p}\right).$$

where *a* indexes the attention head and *t* indexes the token position within that head; ${\mathrm{bp}}_{a,t}$ is the combined bucket–position identifier for the query at (a,t); $b_{a,t}$ is the bucket number assigned to that query; $\tau_{a,t}$ is the sequence-position index of the query; and $N_{p}$ is the sequence length, which is equal to the number of temporal patches defined in Section 3.2.

To place tokens within each bucket in their original sequence order, the identifiers ${\mathrm{bp}}_{a,t}$ are sorted while retaining the pre-sort position indices:

(16) $${\tilde{\mathrm{bp}}}_{a,t},\;\pi_{a,t}=\mathrm{sort}\left({\mathrm{bp}}_{a,t},\;\tau_{a,t}\right),$$

where ${\tilde{\mathrm{bp}}}_{a,t}$ is the sorted combination identifier and $\pi_{a,t}$ is the original position index retained after sorting.

The sorted query matrix $\tilde{Q}$ is obtained by reordering the original query matrix Q according to the within-bucket position indices:

(17) $$\begin{array}{cc} st_{a,t} & =\pi_{a,t}\;\mathrm{mod}\;N_{p}, \end{array}$$

(18) $$\begin{array}{cc} \tilde{Q} & =\mathrm{index}\text{\_}\mathrm{select}(Q,\;st). \end{array}$$

where $st_{a,t}$ is the within-bucket sequence position and $\tilde{Q}$ contains the reordered query vectors.

Finally, the attention output within each hash bucket is computed as

(19) $$o_{i}=\sum_{j\in B_{i}}\exp\;\left(q_{i}\cdot k_{j}-Z\left(i;B_{i}\right)\right)v_{j},$$

where $B_{i}$ denotes the set of token positions that share the same hash bucket as query position *i*; $q_{i}$, $k_{j}$, and $v_{j}$ are the query, key, and value vectors at positions *i* and *j*, respectively; and $Z(i;B_{i})$ is the softmax normalization term.

### 3.4. Training Objective

To classify the output features o, we employ a multi-channel convolutional network as a classifier. We include a margin loss term that maximizes the distance between prototype samples of different categories, thereby strengthening inter-class boundaries and improving the discriminability of the prototype representations in the feature space:

(20) $$\begin{array}{cc} L_{\mathrm{cls},o} & =-\sum_{i}y_{i}\log{\hat{p}}_{i}^{o}, \end{array}$$

(21) $$\begin{array}{cc} L_{\mathrm{margin}} & =\frac{1}{2}\sum_{k\neq k^{\prime }}\max\;\left(0,\;m\;-\;∥p^{k}-p^{k^{\prime }}{∥}^{2}\right). \end{array}$$

where $L_{\mathrm{cls},o}$ is the classification loss computed from the output feature o; $y_{i}$ is the ground-truth label and ${\hat{p}}_{i}^{o}$ is the predicted class probability for the *i*-th sample; $L_{\mathrm{margin}}$ is the inter-class margin loss; $p^{k}$ and $p^{k^{\prime }}$ are prototype vectors of different activity classes ($k\neq k^{\prime }$); and m>0 is the margin threshold.

We additionally compute a classification loss on the prototype samples p to prevent prototype degradation while optimizing the inter-class margin:

(22) $$L_{\mathrm{cls},\mathrm{pt}}=-\sum_{i}y_{i}\log{\hat{p}}_{i}^{pt}.$$

where $L_{\mathrm{cls},\mathrm{pt}}$ is the classification loss of the prototype samples and ${\hat{p}}_{i}^{pt}$ is the corresponding predicted class probability.

The complete training objective combines the three losses above:

(23) $$L_{\mathrm{total}}=L_{\mathrm{cls},o}+\lambda_{1}L_{\mathrm{margin}}+\lambda_{2}L_{\mathrm{cls},\mathrm{pt}},$$

where $\lambda_{1}>0$ and $\lambda_{2}>0$ are weighting coefficients that balance the contribution of the margin loss and the prototype classification loss, respectively.

## 4. Experiments

We evaluated DSHformer on five public sensor-based human activity recognition (HAR) benchmarks and compared it with both HAR-specific baselines and recent Transformer-based time-series models. The experiments are designed to answer four questions: (i) whether DSHformer achieves competitive recognition performance under standard benchmark protocols; (ii) whether each proposed component contributes to the final performance; (iii) whether the model remains effective under subject-disjoint cross-user evaluation; and (iv) how the LSH-based attention module behaves in terms of hyperparameter sensitivity and practical efficiency.

For fair comparison, DSHformer and all baseline models were implemented within the same experimental framework and trained and evaluated under the same data partition for each dataset. In the main benchmark comparison, WISDM and UniMiB-SHAR followed the preprocessed benchmark splits, UCI-HAR followed the official train/test split, PAMAP2 followed the common preprocessed subject/file split, and Opportunity followed the common benchmark split in which the ADL4/ADL5 sessions of subjects S2/S3 were used as held-out test files. For model selection, 10% of the training portion was randomly held out as the validation set, while the original test portion was kept untouched for final evaluation. Unless otherwise stated, the main benchmark comparison and ablation experiments were conducted with a fixed random seed of 42. The additional 10-run stability analysis is introduced after the main benchmark comparison. Therefore, the performance differences reported in the main comparison reflect model-level differences rather than differences in data partitioning.

### 4.1. Dataset and Evaluation Metrics

Datasets.

We used five public sensor-based HAR benchmarks: WISDM [34], UCI-HAR [35], PAMAP2 [36], Opportunity [37], and UniMiB-SHAR (hereafter SHAR) [38]. These datasets cover different sequence lengths, sensor dimensions, and activity categories, providing a broad evaluation of the proposed framework. The dataset statistics are summarized in Table 1.

Evaluation metrics.

We report three metrics: accuracy (Acc), class-average F1-score ($F_{m}$), and weighted F1-score ($F_{w}$). Accuracy measures the overall classification correctness across all classes:

(24) $$\mathrm{Accuracy}=\frac{\sum_{c=1}^{K}\left({\mathrm{TP}}_{c}+{\mathrm{TN}}_{c}\right)}{\sum_{c=1}^{K}\left({\mathrm{TP}}_{c}+{\mathrm{TN}}_{c}+{\mathrm{FP}}_{c}+{\mathrm{FN}}_{c}\right)}.$$

where *K* denotes the number of activity classes, and ${\mathrm{TP}}_{c}$, ${\mathrm{TN}}_{c}$, ${\mathrm{FP}}_{c}$, and ${\mathrm{FN}}_{c}$ denote the true-positive, true-negative, false-positive, and false-negative instances of class *c*, respectively.

The class-average F1-score reflects the recognition performance for each individual activity class regardless of its prevalence in the dataset. It is defined as

(25) $$F_{m}=\frac{1}{K}\sum_{c=1}^{K}\frac{2\cdot {\mathrm{Precision}}_{c}\cdot {\mathrm{Recall}}_{c}}{{\mathrm{Precision}}_{c}+{\mathrm{Recall}}_{c}}.$$

where ${\mathrm{Precision}}_{c}={\mathrm{TP}}_{c}/\left({\mathrm{TP}}_{c}+{\mathrm{FP}}_{c}\right)$ and ${\mathrm{Recall}}_{c}={\mathrm{TP}}_{c}/\left({\mathrm{TP}}_{c}+{\mathrm{FN}}_{c}\right)$ represent the precision and recall for class *c*, respectively.

The weighted F1-score accounts for class imbalance by computing the F1-score for each class and averaging them with weights proportional to the number of samples in each class:

(26) $$F_{w}=\sum_{c=1}^{K}W_{c}\;\frac{2\cdot {\mathrm{Precision}}_{c}\cdot {\mathrm{Recall}}_{c}}{{\mathrm{Precision}}_{c}+{\mathrm{Recall}}_{c}}.$$

where $W_{c}=n_{c}/N$, $n_{c}$ represents the number of samples in class *c*, and *N* denotes the total number of samples in the dataset.

### 4.2. Hyperparameter Selection and Complexity Discussion

Table 2 summarizes the main hyperparameter settings of DSHformer on the five HAR datasets. These hyperparameters include the temporal patch length, the number of decomposition/hash layers, the number of LSH-based attention layers, the number of attention heads, and the hash bucket size.

Here, P_Length denotes the length of each temporal patch used to construct the channel graph. Number_HF denotes the number of stacked decomposition/hash layers and the corresponding number of random projection matrices. Number_AL denotes the number of stacked LSH-based attention layers, Number_AH denotes the number of attention heads in each attention layer, and B_Size denotes the hash bucket size. Since B_Size and Number_HF are important hyperparameters of the LSH-based attention module, we further analyze their robustness under small perturbations in Section 4.6.

The theoretical efficiency advantage of DSHformer mainly comes from the LSH-based attention module. Standard self-attention computes pairwise interactions among all temporal tokens, leading to $O(L^{2})$ attention complexity. In contrast, the LSH-based attention module first assigns temporal tokens to hash buckets through random projections and then computes attention mainly within the selected buckets. Considering the bucket sorting and local attention operations, the theoretical attention complexity is reduced to approximately O(LlogL). Here, *L* denotes the sequence length (corresponding to *T* in Section 3).

For the complete DSHformer model, the computational cost also includes patch-based channel–temporal feature extraction, prototype-guided decomposition, prototype matching, hash-code generation, bucket sorting, and local attention computation. Specifically, during the channel–temporal feature extraction stage, the input sequence of length *L* is divided into temporal patches of length *n*. If the compressed channel dimension is denoted by $d_{c}$, constructing the channel-relation matrix for each patch introduces a cost proportional to $O\;\left(\frac{d_{c}^{2}}{n}L\right)$. The subsequent patch-level temporal modeling operates on $\frac{L}{n}$ tokens.

In the decomposition and LSH-based attention stage, the decomposition network processes the patch-level temporal features with a cost proportional to $O\;\left(N_{\mathrm{hf}}\;\frac{L}{n}\right)$, where $N_{\mathrm{hf}}$ denotes the number of stacked decomposition/hash layers (Number_HF in Table 2). The LSH-based attention module sorts the $\frac{L}{n}$ patch tokens according to their hash buckets, which introduces a sorting cost of $O\;\left(\frac{L}{n}\log\frac{L}{n}\right)$, which is followed by local attention computation within hash buckets.

Therefore, the O(LlogL) term mainly characterizes the asymptotic behavior of the LSH-based attention component. The practical inference cost of the full model can also be affected by the overhead of feature extraction, prototype matching, hashing, and sorting. This distinction is particularly important for short-window datasets such as Opportunity, where the sequence length is only L=30 and the overhead of hashing and sorting may partially offset the theoretical advantage. To avoid overstating the theoretical result, we further report on the full-model latency, memory usage, parameter counts, and attention-core benchmarks in Section 4.7. Table 3 provides a theoretical attention-complexity comparison between DSHformer and representative Transformer variants.

It should be noted that Table 3 compares asymptotic attention complexity rather than full-model wall-clock latency. The latter is evaluated separately in Section 4.7, where we measure inference latency, memory usage, and parameter counts under different sequence-length settings.

As shown in Table 3, DSHformer achieves the same asymptotic attention complexity as Reformer and Informer; the O(LlogL) complexity is therefore not a contribution of this work per se. The novelty lies instead in how LSH-based attention is integrated with the decomposition and prototype-alignment framework. Reformer [5] applies random-projection hashing directly to the input query vectors without any awareness of the underlying feature structure or task-specific semantics. Informer [27] reduces complexity through ProbSparse attention, which selects dominant queries based on a query–key divergence criterion designed for long-sequence forecasting rather than classification. In DSHformer, the random projections are applied to features that have already been decomposed into similar and dissimilar components relative to class-specific prototypes. This decomposition-stable hashing design ensures that tokens sharing similar temporal semantics as identified by the prototype-matching process are consistently assigned to the same hash bucket even after decomposition-induced attenuation of the feature representations. The HAR-tailored integration thus differs from both Reformer and Informer in the structural coupling between prototype-guided decomposition and hash-bucket formation rather than in the asymptotic complexity class itself.

### 4.3. Comparisons with the State of the Art

Table 4 reports the main benchmark comparison on five public HAR datasets. All baselines and DSHformer are evaluated under the same split of each dataset, as described above. Therefore, the results in Table 4 compare model performance under a shared benchmark protocol rather than under different data partitions. The compared methods include HAR-specific models such as TCN [39] and Atten [6], frequency-domain and patch-based time-series models such as FreTS [40] and PatchTST [41], and Transformer-based variants such as Crossformer [42], ETSformer [43], iTransformer [44], Informer [27], Autoformer [23], Transformer [7], and Reformer [5]. For baseline implementation, models with publicly available official or author-released code were implemented based on their released repositories when available. For methods without directly reusable HAR classification code, we reimplemented the corresponding model architectures according to the original papers and adapted only the input embedding and final classification head to the HAR setting. All baselines used the same preprocessed inputs, training/validation/test partitions, optimizer type, batch size, maximum training epochs, early-stopping criterion, and evaluation metrics as DSHformer unless otherwise required by the original model design. Hyperparameters of the baseline models were set according to the recommendations in the original papers or tuned on the validation set under the same protocol. No baseline used additional training data, test-set information, or a different data partition. The three metrics Acc, $F_{m}$, and $F_{w}$ are calculated according to Equations (24)–(26).

The main benchmark results are reported in Table 4, and the 10-run stability analysis is reported separately in Table 5. As shown in Table 4, DSHformer achieves the highest reported accuracy on all five benchmarks with Acc values of 98.6%, 93.7%, 98.4%, 88.5%, and 96.6% on WISDM, UCI-HAR, PAMAP2, Opportunity, and SHAR, respectively. DSHformer also achieves the best or tied-best $F_{m}$ and $F_{w}$ values in most columns. These results indicate that the proposed combination of channel–temporal feature encoding, prototype-guided feature refinement, and LSH-based attention is effective under the adopted benchmark protocols.

Compared with the vanilla Transformer, DSHformer improves the reported accuracy by 2.2, 2.9, 0.3, 2.0, and 2.8 percentage points on WISDM, UCI-HAR, PAMAP2, Opportunity, and SHAR, respectively. The 0.3 pp gain on PAMAP2 is within the observed run-to-run variance (Table 5) and is therefore not claimed as a reliable improvement. Compared with Reformer, the corresponding gains are 3.2, 3.7, 0.5, 1.1, and 9.9 percentage points. The gains are particularly pronounced on WISDM and SHAR, suggesting that the channel–temporal encoder and prototype-guided refinement are effective for extracting discriminative representations from relatively low-dimensional inertial signals.

Beyond the fixed-seed main comparison, Table 5 further summarizes the stability of each model over 10 independent runs. DSHformer achieves the best worst-case accuracy on all five datasets. Its variance is also among the lowest in most settings, although the lowest variance is obtained by other baselines on WISDM, PAMAP2, Opportunity, and SHAR. These results suggest that DSHformer provides a favorable balance between average performance and run-to-run stability; we do not claim that it achieves the lowest variance in every case.

To further address cross-user generalization, Table 6 reports an additional subject-disjoint comparison with representative Transformer-based baselines. In this setting, the subjects used for training, validation, and testing do not overlap. For WISDM, we used five folds over 36 subjects, with test subject groups 1–7, 8–14, 15–21, 22–28, and 29–36, and validation subjects 8–9, 15–16, 22–23, 29–30, and 1–2, respectively. For UCI-HAR and UniMiB-SHAR, we used five folds over 30 subjects with six test subjects and two validation subjects in each fold. For PAMAP2, we used a nine-fold subject-disjoint protocol over subjects 101–109: in each fold, one subject was used as the test subject, the next subject was used as the validation subject, and the remaining seven subjects were used for training; the test/validation pairs were 101/102, 102/103, 103/104, 104/105, 105/106, 106/107, 107/108, 108/109, and 109/101. For Opportunity, we used four folds over subjects 1–4 with test/validation pairs 1/2, 2/3, 3/4, and 4/1.

However, the subject-disjoint result on Opportunity is different from the other four datasets. On this dataset, the Reformer-style sparse-attention baseline obtains the highest Macro-$F_{1}$, while DSHformer performs worse than both Transformer and Reformer-style baselines. This negative result suggests that cross-user generalization remains challenging for very short and high-dimensional sensor sequences, such as Opportunity with sequence length L=30 and 113 sensor axes. We therefore treat the subject-disjoint Opportunity result as an important limitation rather than over-claiming uniform cross-user superiority.

The selected baselines cover complementary modeling strategies. TCN captures multi-scale temporal patterns with dilated convolutions, Atten introduces attention-based regularization for wearable HAR, FreTS models time-series patterns in the frequency domain, and PatchTST uses patch-wise time-series modeling. The Transformer-based models provide representative comparisons for full attention, sparse or efficient attention, frequency-enhanced decomposition, and cross-dimension dependency modeling. The recent AutoHFormer [45], an efficient hierarchical autoregressive Transformer for time-series prediction, was considered but not included as a direct baseline: its design targets causal long-horizon forecasting through hierarchical temporal modeling, intra-segment autoregressive refinement, dynamic windowed attention with exponential decay, and adaptive multi-scale temporal encoding. Its core objective—predicting continuous future values under strict temporal causality—differs fundamentally from the fixed-window single-label classification task in sensor-based HAR. Adapting AutoHFormer to HAR would require replacing its autoregressive forecasting objective and multi-step output head with a discriminative classification head, substantially altering the original model and making any comparison implementation-dependent; we therefore cite AutoHFormer as a relevant recent efficient time-series Transformer and note its faithful adaptation to HAR classification as a direction for future work. Compared with the included methods, DSHformer achieves strong recognition performance under the standard benchmark protocol and competitive subject-disjoint performance on most datasets. The efficiency of the LSH-based attention module and the practical full-model cost are analyzed separately in Section 4.7.

### 4.4. Ablation Studies, Visualization, and Analysis

Table 7 reports the component-level ablation results under the standard benchmark protocol. The Transformer is used as the reference baseline. Ours (MT) keeps only the patch-based channel–temporal feature extraction module. Ours (att) keeps the decomposition network and the LSH-based attention module without the patch-based channel–temporal encoder. Ours (MT + att) combines the channel–temporal encoder with the decomposition-guided LSH-based attention module. Ours (att + $L_{\mathrm{total}}$) further introduces the total training objective, including the prototype-related regularization terms. Ours (full) denotes the complete DSHformer model.

As shown in Table 7, Ours (MT) improves over the vanilla Transformer on WISDM, UCI-HAR, and SHAR, indicating that the patch-based channel–temporal encoder provides an effective representation for wearable sensor signals. On Opportunity, however, Ours (MT) obtains lower accuracy than the Transformer. This result suggests that channel–temporal encoding alone is not sufficient for all datasets especially when the sequence length is short and the sensor dimension is high.

Ours (att) contains the decomposition network and the LSH-based attention module but does not use the proposed patch-based channel–temporal encoder. Its performance is lower than the full model on all five datasets. This indicates that the LSH-based attention module requires informative patch-level representations as input; using the attention and decomposition modules alone cannot fully recover the temporal and cross-channel information needed for HAR.

Ours (MT + att) combines the channel–temporal encoder with the decomposition-guided LSH-based attention module. This variant improves over Ours (MT) on Opportunity in terms of Acc and Fm, reaching 0.882 and 0.816, respectively. It also achieves strong performance on SHAR, with Acc, Fm, and Fw values of 0.958, 0.932, and 0.954, respectively. However, its performance is still lower than Ours (full) on all five datasets, indicating that the complete training objective and prototype-related regularization remain important.

Ours (att + $L_{\mathrm{total}}$) adds the total training objective to the attention-based variant. Compared with Ours (att), this setting improves several metrics, especially on SHAR and Opportunity, which suggests that the prototype-related loss terms help regularize the representation space. Nevertheless, without the patch-based channel–temporal encoder, its performance remains lower than the full model on most datasets.

The full DSHformer model achieves the best overall results in Table 7, confirming that the three main components are complementary: the patch-based encoder provides compact channel–temporal representations, the decomposition-guided LSH-based attention module refines the temporal features, and $L_{\mathrm{total}}$ regularizes both the classifier output and the prototype representations.

To further examine whether prototype alignment contributes to cross-user generalization, Table 8 compares the full model with a variant that disables prototype alignment under the subject-disjoint protocol. In this setting, the training, validation, and test subjects do not overlap. The w/o Prototype variant disables the prototype alignment path and removes the prototype auxiliary classification loss. The full model consistently outperforms w/o Prototype on all five datasets. On average, prototype alignment improves Acc, $F_{m}$, and $F_{w}$ by 0.0267, 0.0308, and 0.0266, respectively.

These subject-disjoint ablation results provide additional evidence that the prototype mechanism is not only beneficial under the standard benchmark protocol but also contributes to unseen-subject evaluation. The improvement in Macro-$F_{1}$ is numerically largest on PAMAP2 and Opportunity (0.0489 and 0.0510, respectively); however, given the large cross-fold standard deviations on these two datasets (see Table 8), these gains should be interpreted with caution and their statistical significance verified. The absolute subject-disjoint performance on Opportunity remains lower than on the other datasets, which is consistent with the observation in Table 6 that short and high-dimensional sensor sequences remain challenging for cross-user generalization.

**Figure 3:**
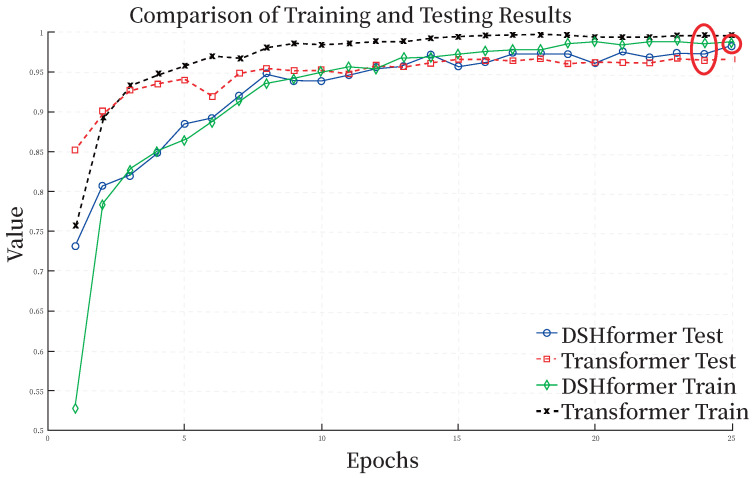
Training vs. testing results: DSHformer vs. Transformer. The red circles highlight the final-epoch testing comparison.

**Figure 4:**
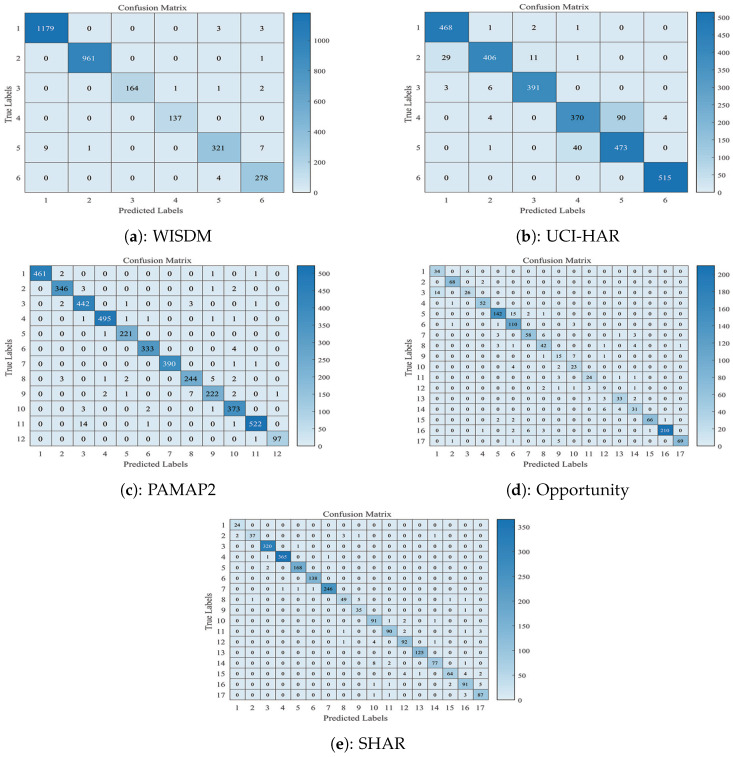
Confusion matrices of DSHformer on five HAR benchmarks.

We further provide qualitative visualizations to support the above quantitative findings. Figure 3 provides an illustrative comparison of the training and testing behavior of the vanilla Transformer and DSHformer. Compared with the baseline Transformer, DSHformer shows a smaller train–test gap in this example, suggesting a reduced tendency to overfit the training data. Figure 4 presents the confusion matrices of DSHformer on the five HAR benchmarks. The diagonal patterns indicate that most samples are correctly classified, while the remaining off-diagonal entries mainly correspond to activities with similar motion patterns.

**Figure 5:**
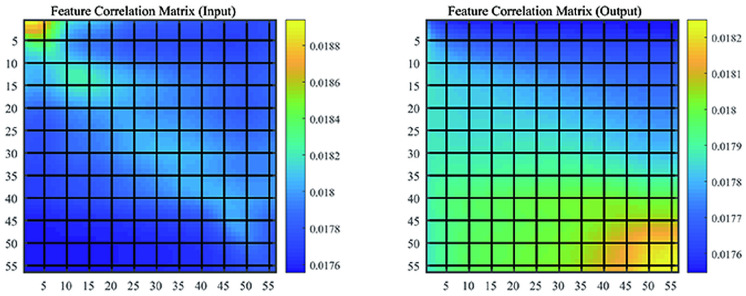
Feature correlation matrices before and after encoding.

The goal of the patch-based channel–temporal encoder is to aggregate related information across sensor channels and temporal patches. Figure 5 visualizes the feature correlation matrices before and after the encoder. The correlation matrix is computed as $xx^{⊤}$, where x denotes the feature matrix and ⊤ denotes the matrix transpose. In the illustrated UCI-HAR example, the input features show relatively weak correlations across time steps before encoding. After channel–temporal encoding, the correlations become more structured, indicating that the encoder aggregates related temporal and channel-wise information into more coherent feature representations.

**Figure 6:**
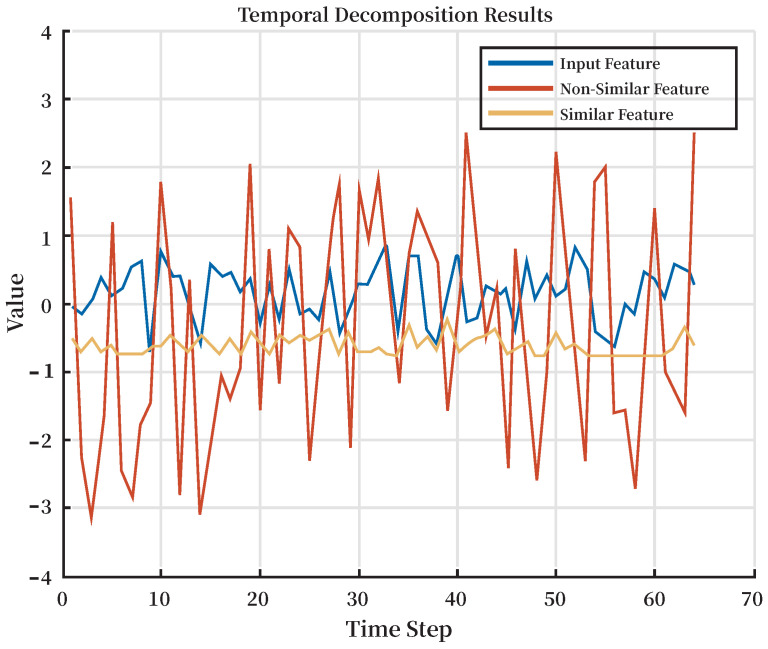
Visualization of temporal decomposition results.

Figure 6 visualizes the temporal decomposition results on a representative variable channel from UCI-HAR. The decomposition network is designed to separate similar and dissimilar components between the input features and the selected prototype features. In the visualization, the similar component exhibits smoother temporal variations, whereas the dissimilar component contains more oscillatory patterns. This qualitative result is consistent with the intended role of the decomposition module: preserving shared activity-related patterns while separating residual or less aligned information.

**Figure 7:**
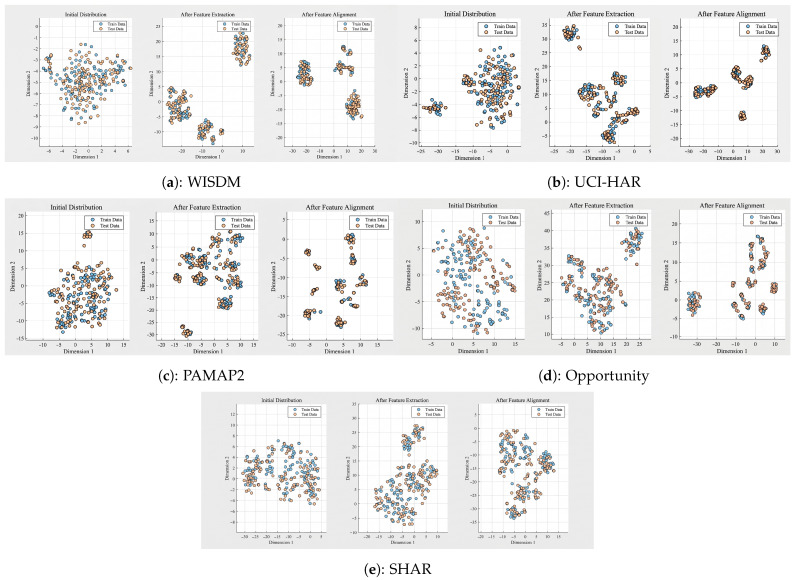
t-SNE visualization of data distributions for DSHformer.

As shown in Figure 7, we further visualize the t-SNE distributions of training and testing samples from the five datasets before model input, after feature extraction, and after prototype-guided alignment. Before model processing, the samples are more dispersed and class boundaries are less clear. After feature extraction, samples of the same class tend to form more compact clusters. After prototype-guided alignment, the training and testing distributions become visually more comparable in several datasets, suggesting that the proposed alignment mechanism can reduce part of the distribution discrepancy. This visualization should be interpreted as qualitative evidence, while the quantitative subject-disjoint results provide a stricter evaluation of cross-user generalization.

**Figure 8:**
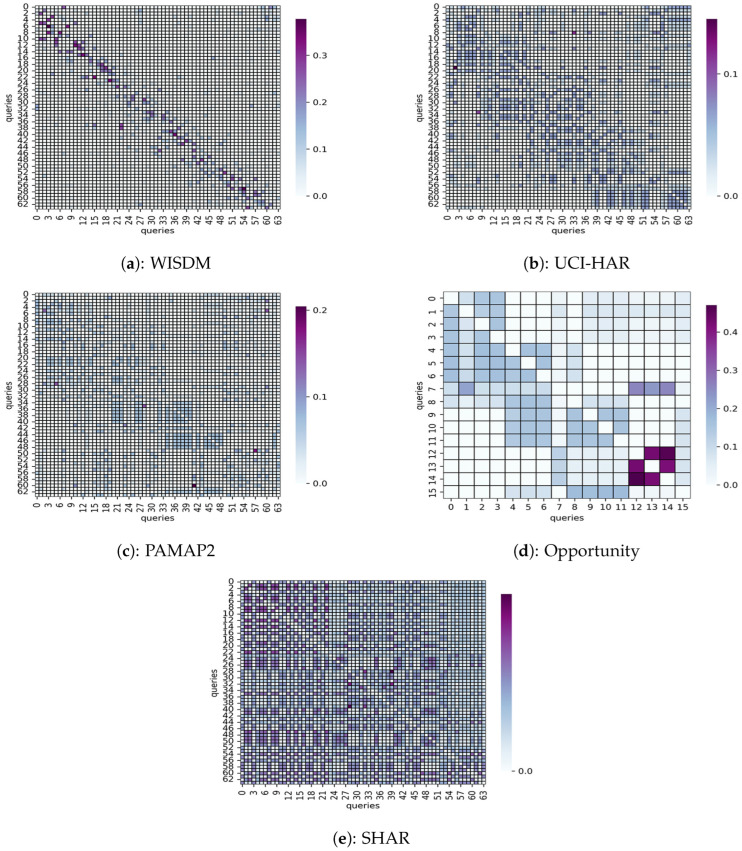
Visualization of LSH-based attention matrices.

As shown in Figure 8, we visualize the attention matrices produced by the LSH-based attention module. Compared with the full attention matrix of the vanilla Transformer, the LSH-based attention matrix has a different sparsity pattern because attention is computed only among tokens assigned to the same hash bucket. As discussed in Section 3.3, the implementation sets $k_{j}=q_{j}/∥q_{j}∥$, which guarantees $H\left(k_{j}\right)=H\left(q_{j}\right)$; the diagonal entries of the visualized LSH-based attention matrix are therefore zero. In addition, blank cells in the LSH-based attention matrix indicate token pairs that are not involved in attention computation due to different hash assignments, whereas blank cells in the Transformer attention map mainly correspond to very small attention weights.

The sparsity patterns also vary across datasets. For example, the attention matrix on SHAR is visually denser than that on WISDM. This is consistent with the dataset-specific bucket sizes reported in Table 2: the bucket sizes for WISDM, UCI-HAR, PAMAP2, Opportunity, and SHAR are 2, 8, 8, 4, and 32, respectively. Therefore, larger bucket sizes allow more tokens to participate in local attention within the same bucket, leading to denser attention maps. Overall, Figure 8 illustrates how LSH-based attention reduces unnecessary pairwise computations by restricting attention to tokens with the same hash assignment.

### 4.5. Effect of Feature Extraction Order

**Figure 9:**
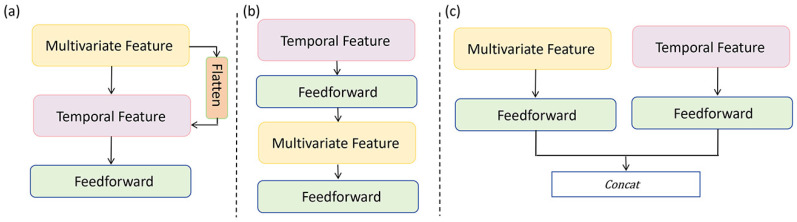
Comparison of model performance with different feature extraction orders. (**a**) Joint feature extraction; (**b**) temporal-first feature extraction; (**c**) channel-first feature extraction.

To further examine the design of the channel–temporal feature encoder, we compare three feature extraction orders: Joint, Temporal-First, and Channel-First. The Joint structure extracts channel and temporal features in parallel, the Temporal-First structure first models temporal patterns and then incorporates channel information, and the Channel-First structure first models inter-channel relationships before temporal feature extraction. Figure 9 provides a visual comparison of the three structures, and Table 9 reports their quantitative results on the five HAR benchmarks in terms of Acc, $F_{m}$, and $F_{w}$.

As shown in Table 9, the Joint structure achieves competitive performance on all five datasets, with accuracies of 97.3%, 92.4%, 98.0%, 86.1%, and 95.3% on WISDM, UCI-HAR, PAMAP2, Opportunity, and SHAR, respectively. This result indicates that jointly considering channel and temporal information is beneficial for HAR, but direct joint modeling does not consistently provide the best performance across datasets.

The Temporal-First structure improves over the Joint structure on WISDM, Opportunity, and SHAR in terms of accuracy, reaching 98.5%, 86.7%, and 95.5%, respectively. However, its performance decreases on UCI-HAR and PAMAP2. This suggests that extracting temporal patterns before channel interaction can be effective for some datasets, but it may also weaken the modeling of inter-channel relationships when channel correlations are important for activity discrimination.

The Channel-First structure achieves the best performance across all five datasets. It obtains accuracies of 98.6%, 93.7%, 98.4%, 88.5%, and 96.6% on WISDM, UCI-HAR, PAMAP2, Opportunity, and SHAR, respectively. It also achieves the highest $F_{m}$ and $F_{w}$ values in all reported settings. These results support the encoder design used in DSHformer, where inter-channel relationships are modeled before patch-level temporal refinement. This order allows the model to first capture dependencies among sensor axes or modalities and then use the resulting channel-aware representations for temporal modeling.

Overall, the comparison in Table 9 shows that the order of feature extraction has a clear influence on HAR performance. The Channel-First design provides the most consistent results across datasets with different numbers of sensor axes and sequence lengths. This observation is consistent with the characteristics of wearable sensor data, where multiple channels often describe complementary aspects of the same physical motion. Modeling these channel relationships before temporal aggregation can therefore provide more informative inputs for subsequent sequence modeling.

### 4.6. Sensitivity Analysis of LSH Hyperparameters

To analyze the robustness of the LSH-based attention module, we conduct a sensitivity study on two important hyperparameters: the hash bucket size B_Size and the number of hash/decomposition layers Number_HF. These two hyperparameters control the granularity of hash-based token grouping and the number of random projection rounds. Since overly small buckets may restrict useful token interactions and overly large buckets may reduce the sparsity benefit of LSH-based attention, it is important to examine whether the model remains stable under small perturbations.

We perform this analysis on UCI-HAR and Opportunity. UCI-HAR has a moderate sequence length (L=128) and relatively low sensor dimension, whereas Opportunity has a very short sequence length (L=30) and a high sensor dimension of 113 axes. These two datasets therefore provide representative settings for evaluating the sensitivity of the LSH-based attention module under different temporal and channel conditions. Table 10 reports the results.

As shown in Table 10, DSHformer is relatively stable on UCI-HAR under small changes of B_Size and Number_HF. Across the tested settings, the Macro-$F_{1}$ ranges from 0.8924 to 0.9181, and the Acc ranges from 0.8951 to 0.9192. The best UCI-HAR result is obtained when B_Size is 4 and Number_HF is 2, but the other settings also remain close to this value. This indicates that the model is not highly sensitive to a single fixed bucket size on UCI-HAR.

The behavior on Opportunity is more sensitive. The best Macro-$F_{1}$ is obtained when B_Size is 4 and Number_HF is 1, while increasing Number_HF to 2 under the same bucket size leads to a clear performance drop. This is consistent with the characteristics of Opportunity: the sequence length is very short (L=30), while the sensor dimension is high. Under such a setting, additional hash/decomposition rounds may introduce extra grouping and sorting overhead without providing enough long-range temporal context to compensate for it.

Overall, the sensitivity results suggest that DSHformer is reasonably robust to small hyperparameter perturbations on moderate-length sensor sequences, while short-window and high-dimensional datasets require a more careful selection of B_Size and Number_HF. This also motivates future work on adaptive or dataset-agnostic bucket/hash selection strategies.

### 4.7. Efficiency Analysis

To further examine whether the theoretical complexity advantage of LSH-based attention leads to practical efficiency gains, we conduct two types of efficiency experiments. First, we compare the full-model inference cost of DSHformer and the vanilla Transformer under real HAR dataset configurations. Second, we isolate the attention operation and compare full attention with bucketed attention under different sequence lengths. This design allows us to distinguish between the theoretical attention-level advantage and the practical wall-clock cost of the complete HAR model. We include the vanilla Transformer in the full-model benchmark; a full-model wall-clock comparison with the original Reformer architecture is not included because the Reformer-style baseline used in our HAR experiments is a sparse-attention adaptation of the pipeline rather than a complete reproduction of the original Reformer (see Table 6 footnote), and the attention-core benchmark captures the relevant efficiency difference at the attention-mechanism level.

Table 11 reports the full-model efficiency comparison under the real configurations of Opportunity and UCI-HAR. The results show that the current full DSHformer implementation has more parameters, higher inference latency, and higher peak memory usage than the vanilla Transformer. This is especially clear on Opportunity, where the sequence length is only L=30. Although the LSH-based attention module reduces the theoretical attention complexity, the complete model also includes channel–temporal feature extraction, prototype matching, decomposition, hashing, bucket sorting, and local attention computation. Therefore, the attention-level complexity advantage does not directly imply full-model latency advantage, particularly for short-window datasets.

Table 12 reports the attention-core benchmark under controlled sequence lengths. For short sequences (L=32, 64, and 128), bucketed attention does not provide a latency advantage because the overhead of hashing, bucket assignment, and sorting is comparable to or larger than the cost saved from sparse attention computation. However, as the sequence length increases, the advantage of bucketed attention becomes clear. At L=256, bucketed attention is 2.47× faster and uses 3.32× less memory than full attention. At L=1024, it is 12.48× faster and reduces memory usage by 14.75×.

These two efficiency experiments lead to a more precise interpretation of the proposed method. The LSH-based attention core provides clear speed and memory advantages for longer sequences, which supports the theoretical O(LlogL) attention-complexity analysis. However, the current full DSHformer implementation still incurs additional overhead from feature extraction, prototype-guided decomposition, prototype matching, hashing, and sorting. Therefore, DSHformer should not be interpreted as being faster than the vanilla Transformer in all practical settings. Instead, the empirical results show that the attention-level advantage becomes more useful as the sequence length increases, while short-window datasets such as Opportunity remain affected by implementation overhead.

## 5. Conclusions

We presented DSHformer, which is a Transformer-based framework for sensor-based human activity recognition that jointly considers compact channel–temporal representation learning, prototype-guided feature refinement, and efficient sequence modeling. The framework integrates three complementary components: (i) a low-parameter patch-based graph-attention encoder that jointly models inter-channel correlations and temporal dynamics; (ii) a prototype pool coupled with a multi-layer decomposition network that refines input representations through prototype matching and is further regularized by an inter-class margin loss; and (iii) a decomposition-guided LSH-based attention mechanism tailored for HAR, which couples prototype-guided feature decomposition with locality-sensitive hashing to ensure that semantically related tokens remain grouped throughout the attention computation, retaining the O(LlogL) attention-level complexity of LSH-based attention. Extensive experiments on five public benchmarks—WISDM, UCI-HAR, PAMAP2, Opportunity, and SHAR—show that DSHformer achieves competitive or superior performance compared with HAR-specific baselines and recent Transformer variants under the adopted benchmark protocols. Ablation studies further validate the contribution of the main components, and additional analyses examine subject-disjoint generalization, LSH hyperparameter sensitivity, and practical efficiency under different sequence lengths.

Limitations.

Despite the promising results, this paper has several limitations that warrant further investigation. First, the main benchmark experiments follow the standard or commonly used evaluation protocols of the public HAR datasets, and all baseline models are evaluated under the same data splits for fair comparison. Nevertheless, such benchmark protocols do not always fully reflect the more challenging unseen-user deployment scenario. We therefore added subject-disjoint experiments to further examine cross-user generalization, but more exhaustive LOSO evaluation across all possible subject folds remains an important direction for future work. Second, the prototype pool grows linearly with the number of activity classes, so scalability to fine-grained or open-set HAR scenarios with dozens of classes has not been systematically studied. Third, although the LSH-based attention module reduces the theoretical complexity of attention computation to O(LlogL), the practical inference cost of the full model also includes feature extraction, prototype matching, hashing, bucket sorting, and other implementation overhead. Therefore, the empirical efficiency gain is sequence-length dependent: it is more evident for relatively long sequences, whereas for very short windows such as Opportunity (L=30), the overhead of hashing and sorting can partially offset the theoretical advantage. Fourth, our current evaluation is mainly based on inertial-sensor modalities, such as accelerometers, gyroscopes, and magnetometers. Extending DSHformer to richer physiological or multimodal sensing settings, such as sEMG, PPG, or heterogeneous sensor fusion, requires further architectural adaptation. Finally, the hyperparameters of the LSH-based attention module, particularly the bucket size and the number of hash rounds, are currently selected at the dataset level. Although the added sensitivity analysis shows that the model is reasonably stable under small perturbations in several settings, an adaptive or dataset-agnostic bucket/hash selection strategy would make the framework easier to deploy in practice.

Future Work.

Building on the above limitations, several directions deserve further exploration. First, for cross-user and cross-device HAR, future work will conduct more exhaustive LOSO evaluation and investigate domain adaptation techniques to improve robustness across different users, devices, and deployment environments. Second, for real-time and low-latency inference, we plan to explore model compression, quantization, and hardware-aware optimization of the LSH-based attention module and the prototype matching process. Third, for irregularly sampled and multimodal time series, future work will investigate adaptive interpolation, continuous-time sequence modeling, and modality-specific fusion strategies. Overall, this paper provides a practical step toward accurate and efficient sensor-based HAR while also identifying several important directions for improving cross-user robustness and deployment efficiency.

## Figures and Tables

**Table 1:** Dataset statistics.

Dataset	Number of Samples	Time Series Length	Number of Axes	Number of Activities
WISDM	7678	200	3	6
UCI-HAR	7352	128	9	6
PAMAP2	17,912	171	36	12
Opportunity	14,719	30	113	17
SHAR	9409	151	3	17

**Table 2:** Hyperparameter settings of DSHformer on the five datasets.

Dataset	P_Length	Number_HF	Number_AL	Number_AH	B_Size
WISDM	4	6	2	8	2
UCI-HAR	2	2	1	8	8
PAMAP2	2	1	2	8	8
Opportunity	2	1	2	8	4
SHAR	2	4	2	8	32

**Table 3:** Theoretical attention-complexity comparison with representative Transformer variants.

Method	Crossformer	Informer	Transformer	Reformer	DSHformer
Attention complexity	$O(L^{2})$	O(LlogL)	$O(L^{2})$	O(LlogL)	O(LlogL)

**Table 4:** Baseline comparison on five HAR benchmarks. Each cell reports Acc/$F_{m}$/$F_{w}$. **Bold** indicates the best value in each column; when two methods tie, both are bolded.

Model	WISDM	UCI-HAR	PAMAP2	Opportunity	SHAR
TCN [39]	0.909 /0.873/0.906	0.839/0.834/0.836	0.944/0.935/0.943	0.849/0.787/0.848	0.789/0.730/0.786
Atten [6]	0.942/0.938/0.939	0.933/0.931/0.931	0.983/0.980/0.981	0.876/0.795/0.864	0.930/0.886/0.933
FreTS [40]	0.846/0.788/0.838	0.927/0.925/0.926	0.960/0.954/0.959	0.766/0.678/0.769	0.815/0.748/0.815
PatchTST [41]	0.915/0.829/0.913	0.866/0.868/0.865	0.863/0.868/0.862	0.574/0.445/0.558	0.887/0.846/0.886
Crossformer [42]	0.951/0.947/0.951	0.900/0.897/0.897	0.954/0.949/0.953	0.800/0.729/0.804	0.894/0.859/0.894
ETSformer [43]	0.908/0.863/0.906	0.829/0.804/0.798	0.912/0.897/0.904	0.827/0.757/0.827	0.805/0.745/0.803
iTransformer [44]	0.865/0.805/0.855	0.936/**0.935**/**0.934**	0.968/0.965/0.967	0.802/0.718/0.804	0.894/0.856/0.893
Informer [27]	0.942/0.919/0.939	0.894/0.892/0.892	0.980/0.977/0.979	0.869/0.815/0.869	0.861/0.813/0.857
Autoformer [23]	0.813/0.532/0.769	0.485/0.410/0.393	0.563/0.533/0.503	0.384/0.305/0.375	0.654/0.483/0.633
Transformer [7]	0.964/0.942/0.959	0.908/0.905/0.905	0.981/0.979/0.980	0.865/0.812/0.867	0.938/0.916/0.944
Reformer [5]	0.954/0.952/0.957	0.900/0.899/0.898	0.979/0.976/0.978	0.874/0.819/0.874	0.867/0.811/0.863
**DSHformer (ours) **	**0.986**/**0.979**/**0.985**	**0.937**/**0.935**/**0.934**	**0.984**/**0.981**/**0.982**	**0.885**/**0.833**/**0.886**	**0.966**/**0.947**/**0.965**

**Table 5:** Model stability over 10 independent runs: accuracy variance (lower is better) and worst-case accuracy (higher is better). **Bold** indicates the best value in each column.

Model	WISDM		UCI-HAR		PAMAP2		Opportunity		SHAR	
	Var	Wst	Var	Wst	Var	Wst	Var	Wst	Var	Wst
TCN	1.03 × 10^−5^	0.903	1.86 × 10^−5^	0.835	3.15 × 10^−6^	0.941	3.69 × 10^−5^	0.842	1.38 × 10^−5^	0.786
Atten	2.32 × 10^−5^	0.937	4.97 × 10^−5^	0.926	**5.60 × 10^−8^**	0.979	9.63 × 10^−5^	0.870	2.56 × 10^−5^	0.925
FreTS	**4.26 × 10^−6^**	0.837	3.55 × 10^−5^	0.914	1.01 × 10^−5^	0.954	3.89 × 10^−5^	0.761	1.09 × 10^−4^	0.794
PatchTST	1.87 × 10^−5^	0.905	8.50 × 10^−6^	0.862	3.70 × 10^−5^	0.855	1.16 × 10^−4^	0.552	3.05 × 10^−5^	0.878
Crossformer	3.36 × 10^−5^	0.937	6.21 × 10^−5^	0.879	9.05 × 10^−6^	0.950	7.97 × 10^−5^	0.783	7.42 × 10^−6^	0.877
ETSformer	5.05 × 10^−5^	0.894	3.85 × 10^−4^	0.787	7.06 × 10^−4^	0.862	5.56 × 10^−5^	0.821	1.12 × 10^−5^	0.801
iTransformer	5.62 × 10^−6^	0.863	1.84 × 10^−5^	0.930	2.07 × 10^−6^	0.965	1.52 × 10^−4^	0.723	**1.89 × 10^−6^**	0.894
Informer	1.10 × 10^−5^	0.935	4.98 × 10^−5^	0.884	4.01 × 10^−5^	0.967	9.39 × 10^−4^	0.800	8.88 × 10^−5^	0.845
Autoformer	1.59 × 10^−4^	0.792	6.43 × 10^−4^	0.448	1.41 × 10^−4^	0.548	4.52 × 10^−4^	0.349	1.17 × 10^−4^	0.626
Transformer	8.37 × 10^−6^	0.960	2.01 × 10^−4^	0.871	9.01 × 10^−6^	0.973	1.09 × 10^−5^	0.863	4.33 × 10^−5^	0.935
Reformer	1.66 × 10^−5^	0.945	3.42 × 10^−4^	0.855	8.05 × 10^−5^	0.960	**6.45 × 10^−6^**	0.873	3.84 × 10^−5^	0.857
**DSHformer (ours )**	6.72 × 10^−6^	**0.982**	**1.08 × 10^−5^**	**0.932**	1.40 × 10^−7^	**0.981**	1.71 × 10^−5^	**0.879**	9.76 × 10^−6^	**0.962**

Var: variance of accuracy across 10 runs (lower is better). Wst: worst (minimum) accuracy across 10 runs (higher is better). On WISDM-Var and PAMAP2-Var, the best value is held by another method; DSHformer achieves a favorable overall balance between average performance and stability across datasets.

**Table 6:** Subject-disjoint comparison with representative Transformer-based baselines. Each cell reports Macro-$F_{1}$ as mean ± standard deviation across folds. **Bold** indicates the best value for each dataset.

Dataset	Transformer [7]	Reformer-Style [5]	DSHformer (Ours)
WISDM	0.6941±0.0497	0.7441±0.0498	0.8190±0.0670
UCI-HAR	0.8890±0.0447	0.8991±0.0363	0.9254±0.0440
PAMAP2	0.6398±0.1435	0.5919±0.2335	0.7418±0.0886
Opportunity	0.3938±0.0506	0.4228±0.0774	0.3057±0.0192
SHAR	0.3263±0.0431	0.3631±0.0488	0.6303±0.0463

The Reformer-style model denotes a sparse-attention baseline implemented under the same HAR pipeline and subject-disjoint folds rather than a direct reproduction of the complete original Reformer architecture.

**Table 7:** Ablation study of DSHformer under the standard benchmark protocol. Each cell reports Acc/$F_{m}$/$F_{w}$ components separately by column.

Model	WISDM			UCI-HAR			PAMAP2			Opportunity			SHAR		
	Acc	$F_{m}$	$F_{w}$	Acc	$F_{m}$	$F_{w}$	Acc	$F_{m}$	$F_{w}$	Acc	$F_{m}$	$F_{w}$	Acc	$F_{m}$	$F_{w}$
Transformer	0.964	0.942	0.959	0.908	0.905	0.905	0.981	0.979	0.980	0.865	0.812	0.867	0.938	0.916	0.944
Ours (MT)	0.983	0.964	0.978	0.936	0.924	0.931	0.981	0.979	0.981	0.849	0.798	0.832	0.954	0.937	0.949
Ours (att)	0.917	0.891	0.913	0.901	0.897	0.901	0.979	0.976	0.978	0.848	0.790	0.849	0.876	0.847	0.875
Ours (MT + att)	0.985	0.971	0.981	0.934	0.925	0.929	0.980	0.978	0.979	0.882	0.816	0.828	0.958	0.932	0.954
Ours (att + $L_{\mathrm{total}}$)	0.925	0.895	0.923	0.901	0.900	0.908	0.983	0.976	0.976	0.849	0.812	0.839	0.901	0.879	0.901
Ours (full)	**0.986**	**0.979**	**0.985**	**0.937**	**0.935**	**0.934**	**0.984**	**0.981**	**0.982**	**0.885**	**0.833**	**0.886**	**0.966**	**0.947**	**0.965**

**Table 8:** Subject-disjoint ablation of prototype alignment. Each cell reports mean ± standard deviation across subject-disjoint folds. **Bold** indicates the better result between the two variants.

Dataset	Model	Acc	$F_{m}$	$F_{w}$
WISDM	w/o Prototype	0.8397±0.0427	0.7927±0.1021	0.8434±0.0453
WISDM	Full	0.8551±0.0243	0.8190±0.0670	0.8627±0.0193
UCI-HAR	w/o Prototype	0.9166±0.0567	0.9159±0.0594	0.9147±0.0582
UCI-HAR	Full	0.9228±0.0455	0.9254±0.0440	0.9233±0.0435
PAMAP2	w/o Prototype	0.7444±0.1321	0.6929±0.1262	0.7226±0.1533
PAMAP2	Full	0.7930±0.0975	0.7418±0.0886	0.7722±0.1219
Opportunity	w/o Prototype	0.4040±0.0709	0.2547±0.0479	0.3831±0.0635
Opportunity	Full	0.4463±0.0450	0.3057±0.0192	0.4191±0.0438
SHAR	w/o Prototype	0.7215±0.0431	0.6119±0.0333	0.7197±0.0377
SHAR	Full	0.7425±0.0390	0.6303±0.0463	0.7392±0.0404
Average gain	Full − w/o Prototype	+0.0267	+0.0308	+0.0266

**Table 9:** Feature extraction order comparison.

Model	WISDM			UCI-HAR			PAMAP2			Opportunity			SHAR		
	Acc	$F_{m}$	$F_{w}$	Acc	$F_{m}$	$F_{w}$	Acc	$F_{m}$	$F_{w}$	Acc	$F_{m}$	$F_{w}$	Acc	$F_{m}$	$F_{w}$
Joint	0.973	0.958	0.971	0.924	0.924	0.923	0.980	0.978	0.979	0.861	0.784	0.859	0.953	0.927	0.952
Temporal-First	0.985	0.974	0.978	0.921	0.920	0.920	0.974	0.971	0.973	0.867	0.812	0.867	0.955	0.927	0.954
Channel-First	0.986	0.979	0.985	0.937	0.935	0.934	0.984	0.981	0.982	0.885	0.833	0.886	0.966	0.947	0.965

**Table 10:** Sensitivity analysis of B_Size and Number_HF on UCI-HAR and Opportunity. Each row reports the test performance of DSHformer under one hyperparameter setting. **Bold** indicates the best Macro-$F_{1}$ for each dataset.

Dataset	B_Size	Number_HF	Acc	$F_{m}$	$F_{w}$
UCI-HAR	4	1	0.8982	0.8968	0.8967
UCI-HAR	4	2	**0.9192**	**0.9181**	**0.9186**
UCI-HAR	8	1	0.8951	0.8924	0.8927
UCI-HAR	8	2	0.9114	0.9114	0.9116
UCI-HAR	16	1	0.8955	0.8955	0.8952
UCI-HAR	16	2	0.9104	0.9104	0.9102
Opportunity	2	1	0.3926	0.2720	0.3805
Opportunity	2	2	**0.4387**	0.2800	0.4022
Opportunity	4	1	0.4304	**0.2999**	**0.4169**
Opportunity	4	2	0.3139	0.2443	0.3287

**Table 11:** Full-model efficiency comparison under real HAR dataset configurations. Latency is measured per batch and per sample during inference.

Dataset	Model	Seq. Len.	Params	Latency/Batch	Latency/Sample	Peak Memory
Opportunity	Transformer	30	0.21 M	0.428 ms	0.0067 ms	50.93 MB
Opportunity	DSHformer	30	9.03 M	45.554 ms	0.7118 ms	214.89 MB
UCI-HAR	Transformer	128	0.23 M	1.163 ms	0.0182 ms	42.40 MB
UCI-HAR	DSHformer	128	2.10 M	60.439 ms	0.9444 ms	133.15 MB

**Table 12:** Attention-core benchmark under different sequence lengths. The benchmark isolates the attention operation and compares full attention with bucketed attention.

Seq. Len.	Full Lat.	Bucketed Lat.	Speedup	Full Mem.	Bucketed Mem.	Mem. Red.
32	0.068 ms	0.069 ms	0.99×	10.13 MB	10.13 MB	1.00×
64	0.092 ms	0.114 ms	0.81×	14.13 MB	12.13 MB	1.16×
128	0.156 ms	0.216 ms	0.72×	28.13 MB	16.13 MB	1.74×
256	1.013 ms	0.411 ms	2.47×	80.13 MB	24.13 MB	3.32×
512	3.830 ms	0.608 ms	6.30×	280.13 MB	40.13 MB	6.98×
1024	15.219 ms	1.219 ms	12.48×	1064.13 MB	72.13 MB	14.75×

## Data Availability

All datasets used in this paper are publicly available. The WISDM dataset can be accessed at https://www.cis.fordham.edu/wisdm/dataset.php, (accessed on 14 July 2025); the UCI-HAR dataset at https://archive.ics.uci.edu/dataset/240/human+activity+recognition+using+smartphones, (accessed on 14 July 2025); the PAMAP2 dataset at https://archive.ics.uci.edu/dataset/231/pamap2+physical+activity+monitoring, (accessed on 6 November 2025); the Opportunity dataset at https://archive.ics.uci.edu/dataset/226/opportunity+activity+recognition, (accessed on 6 November 2025); and the UniMiB-SHAR dataset at https://www.dropbox.com/scl/fi/g5ig8nw9qqd253dz8woax/UniMiB-SHAR.zip?rlkey=o0ltu8ivrr9rsfvdhr1bjv3cc&e=1&dl=0, (accessed on 21 December 2025). The source code of DSHformer is available from the corresponding author upon reasonable request.

## Abbreviations

The following abbreviations are used in this manuscript:

HAR	Human Activity Recognition
LSH	Locality-Sensitive Hashing
DSH	Decomposition and Locality-Sensitive Hashing
CNN	Convolutional Neural Network
RNN	Recurrent Neural Network
LSTM	Long Short-Term Memory
TCN	Temporal Convolutional Network
GNN	Graph Neural Network
MLP	Multi-Layer Perceptron
t-SNE	t-Distributed Stochastic Neighbor Embedding
Acc	Accuracy
$F_{m}$	Class-Average F1-Score
$F_{w}$	Weighted F1-Score
WISDM	Wireless Sensor Data Mining (dataset)
UCI-HAR	UCI Human Activity Recognition (dataset)
PAMAP2	Physical Activity Monitoring for Aging People 2 (dataset)
UniMiB-SHAR	UniMiB Smartphone-based HAR (dataset)
